# SERPINH1 is a Potential Prognostic Biomarker and Correlated With Immune Infiltration: A Pan-Cancer Analysis

**DOI:** 10.3389/fgene.2021.756094

**Published:** 2022-01-04

**Authors:** Yu Wang, Weigang Gu, Weiwei Wen, Xiaofeng Zhang

**Affiliations:** ^1^ Department of Gastroenterology, Affiliated Hangzhou First Peopleʼs Hospital, Zhejiang University School of Medicine, Hangzhou, China; ^2^ Hangzhou Institute of Digestive Diseases, Hangzhou, China; ^3^ Key Laboratory of Integrated Traditional Chinese and Western Medicine for Biliary and Pancreatic Diseases of Zhejiang Province, Hangzhou, China; ^4^ Department of Dermatology, Third People’s Hospital of Hangzhou, Hangzhou, China

**Keywords:** SERPINH1, prognosis, immune infiltrate, pan-cancer, biomarker

## Abstract

**Background:** Serpin peptidase inhibitor clade H, member 1 (SERPINH1) is a gene encoding a member of the serpin superfamily of serine proteinase inhibitors. The upregulated of SERPINH1 was associated with poor prognosis in breast cancer, stomach adenocarcinoma, and esophageal carcinoma. However, the role of SERPINH1 in pan-cancer is largely unexplored.

**Methods:** SERPINH1 expression and the correlation with prognosis in human pan-cancer were analyzed by the Cancer Genome Atlas and the Genotype-Tissue Expression dataset. Pearson correlation analysis was applied to evaluate the role of SERPINH1 expression in tumor mutation burden (TMB), microsatellite instability (MSI), mismatch repair (MMR), DNA methyltransferase, and common immunoregulators. Spearman’s correlation test was used to analysis SERPINH1 expression in tumor immune infiltration and infiltrating immune cells via the Tumor Immune Evaluation Resource database. Furtherly, immunohistochemistry staining of SERPINH1 was acquired from the Human Protein Atlas database for validation.

**Results:** SERPINH1 was abnormally expressed in fourteen cancers. The high expression of SERPINH1 significantly reduced the overall survival (OS), disease-specific survival, and progression free interval in eleven cancers. Moreover, SERPINH1 expression was correlated with MMR, MSI, TMB, and DNA methylation in multiple types of cancer. Also, SERPINH1 expression showed strong association with immunoregulators and immune checkpoint markers in testicular germ cell tumors, brain lower grade glioma (LGG), pheochromocytoma and paraganglioma. In addition, SERPINH1 expression was related to immune cell infiltration in multiple cancers, particularly in breast invasive carcinoma, LGG, and liver hepatocellular carcinoma. The result of immunohistochemistry verification shown that SERPINH1 staining was higher in tumor samples than in normal tissue in colon adenocarcinoma, head and neck squamous cell carcinoma, kidney renal papillary cell carcinoma and cervical squamous cell carcinoma, which was consistent with the result of OS.

**Conclusion:** Overall, these results indicate that SERPINH1 may serve as an important prognostic biomarker and correlate with tumor immunity in human pan-cancer.

## Introduction

Cancer is a major public health problem and the second leading cause of mortality around the world (Beheshtirouy et al., 2021). In the treatment of cancer, immune response, protein dysfunction, specific gene mutations, disorders of intracellular signal transduction pathways had been targeted in the past 2 decades (Yeger et al., 2021). Among them, tumor immunotherapy, as a new type of treatment, has played an anti-tumor effect through immune regulation, and shown significant clinical effects (Dashti et al., 2016; Shamseddine et al., 2021). In addition, accumulating evidence indicates that the tumor growth, development and patient prognostic outcomes are related to immune infiltration, and the research on tumor immune mechanism is particularly important (Kamensek et al., 2021).

Serpin peptidase inhibitor, clade H, member 1 (SERPINH1) encodes a member of the serpin superfamily of serine proteinase inhibitors. SERPINH1 is localized to the endoplasmic reticulum and plays a role in collagen biosynthesis as a collagen-specific molecular chaperone. Abnormal expression of this gene may be a marker for cancer, and the nucleotide polymorphisms in this gene may be associated with preterm birth caused by preterm premature rupture of membranes.

The role of SERPINH1 in cancer is largely unexplored, and its functions appear to be inconsistent. For example, SERPINH1 has been reported to be upregulated, and associated with poor prognosis in breast cancer, stomach adenocarcinoma (STAD), and esophageal carcinoma (ESCA) (Lee et al., 2016; Strack et al., 2020; and Yang et al., 2020). Suppression of SERPINH1 expression with short interfering RNAs has been shown significantly inhibit cell proliferation, migration, and invasion in cervical squamous cell carcinoma (Yamamoto et al., 2013). In contrast, in human neuroblastoma, the aberrant methylation of the promoter CpG island makes the expression level of SERPINH1 relatively low (Yang et al., 2004). Therefore, the molecular mechanism through which SERPINH1 exerts its function and its value for prognostic diagnosis need further study.

In the present study, we systematically analyzed 33 types of cancer to investigate the correlation between SERPINH1 expression and the patients’ prognosis. Moreover, this study totally explored the correlation of SERPINH1 with immunoregulators, immune checkpoints, and tumor-infiltrating immune cells in 33 tumor microenviroments. Taken together, our results suggest that SERPINH1 affects the prognosis of patients and is associated with immune infiltration in many cancers, especially in brain lower grade glioma (LGG).

## Meterials and Methods

### Data Source

Transcriptome data and clinical information for patients of 33 types of cancers were downloaded from the TCGA data portal (http://cancergenome.nih.gov/), and the Genotype-Tissue Expression (GTEx) portal. 33 cancer types including ACC, adrenocortical carcinoma; BLCA, bladder urothelial carcinoma; BRCA, breast invasive carcinoma; CESC, cervical squamous cell carcinoma; CHOL, cholangiocarcinoma; COAD, colon adenocarcinoma; DLBC, lymphoid neoplasm diffuse large B-cell lymphoma; GBM, glioblastoma multiforme; HNSC, head and neck squamous cell carcinoma; KICH, kidney chromophobe; KIRC, kidney renal clear cell carcinoma; KIRP, kidney renal papillary cell carcinoma; LAML, acute myeloid leukemia; LIHC, liver hepatocellular carcinoma; LUAD, lung adenocarcinoma; LUSC, lung squamous cell carcinoma; MESO, mesothelioma; OV, ovarian serous cystadenocarcinoma; PAAD, pancreatic adenocarcinoma; PCPG, pheochromocytoma and paraganglioma; PRAD, prostate adenocarcinoma; READ, rectum adenocarcinoma; SARC, sarcoma; SKCM, skin cutaneous melanoma; TGCT, testicular germ cell tumors; THCA, thyroid carcinoma; THYM, thymoma; UCEC, uterine corpus endometrial carcinoma; UCS, uterine carcinosarcoma; UVM, uveal melanoma.

### Analysis of SERPINH1 Expression in Human Pan-Carcer

The RNA sequencing data in TPM (transcripts per million reads) format were downloaded from TCGA and GTEx portal and processed by the Toil (Vivian et al., 2017). SERPINH1 expression data were normalized by log2 conversion. The Wilcoxon rank sum test was for comparing two independent samples, and Wilcoxon signed rank test was for paired sample.

### Analysis of Survival and Prognosis

The pan-cancer samples were divided into SERPINH1 high and SERPINH1 low expression groups based on the minimum *p*-value approach (Kouyama et al., 2019). The Kaplan–Meier survival curves were utilized to exhibit the correlation of SERPINH1 expression with the prognosis of patients’ overall survival (OS), disease-specifific survival (DSS), progression-free interval (PFI).

### Analysis of Biological Function and Pathway

Interaction network analysis was obtained by employing STRING v11.5 database (http: //string-db.org/), keeping default parameters. The topological properties of the PPI network included average shortest path length, betweenness centrality, closeness centrality, degree, eccentricity, neighborhood connectivity, radiality, stress, and topological coefficient. Gene ontology (GO) and Kyoto Encyclopedia of Genes and Genomes (KEGG) enrichment analysis were performed to identify significant pathways via the “cluster Profiler” package in R (Yu et al., 2012).

### Analysis of SERPINH1 Genetic Alteration and Correlations With TMB and MSI

We applied the cBioPortal website (https://www.cbioportal.org/) to query the genetic variation characteristics of SERPINH1 (Cerami et al., 2012; Gao et al., 2013). The correlations of SERPINH1 expression with TMB and MSI in human pan-cancer were based on the TCGA pan-cancer atlas, and analyzed by spearman’s rank correlation method.

### Analysis of MMR Gene Mutation and DNA Methylation

Mismatch repair (MMR) is a post-replicative repair mechanism that can repairs errors in DNA replication and attenuate chromosomal rearrangements, and is closely related to tumorigenesis (Iyer et al., 2015; Baretti and Le, 2018). In this study, the expression levels of MMR genes (MLH1, MSH2, MSH6, EPCAM, and PMS2) in human pan-cancer were obtained from the TCGA database. The correlation between MMR genes and SERPINH1 expression level were analyzed by pearson’s correlation method. Another epigenetic factor affecting gene expression is DNA methylation. We also evaluated the correlation of four methyltransferases (DNMT1, DNMT2, DNMT3A, and DNMT3B) with SERPINH1 expression by the pearson’s correlation method.

### Analysis the Correlation Between SERPINH1 and Immunoregulators

The relationship between SERPINH1 and immunomodulators (such as immunostimulators, immunoinhibitors, MHC molecules, TILs, receptors, and chemokines) across multiple types of human cancer were evaluated through the TISIDB portal (http://cis.hku.hk/TISIDB/). The correlation analysis was performed to explore the relationship between SERPINH1 and 47 common immune checkpoint genes. To explore the prognostic value of SERPIHN1 on immunotherapy, we analyzed the relationship between SERPINH1 and immunotherapy targets (PD-1, PD-L1, PD-L2, and CTLA4). We utilized the Tumor Immune Estimation Resource (TIMER, https://cistrome.shinyapps.io/timer/) database to explore potential associations between SERPINH1 and the infiltrating immune cells across human cancers.

### Analysis of Immune Infiltration

The TISIDB web portal (http://cis.hku.hk/TISIDB/index.php) was applied to explore the association between SERPINH1 and multiple immune regulatory factors across multiple cancer types (Ru et al., 2019). The TIMER database (https://cistrome.shinyapps.io/timer) was used to analyze the correlation between SERPINH1 expression and immune cell infiltration level across human cancers (Li et al., 2017). Spearman correlation analysis was applied to evaluate the correlation between SERPINH1 expression and the scores of multiple immune cells, including B cells, CD4^+^ T cells, CD8^+^ T cells, macrophages, neutrophils, and myeloid denedritic cells.

### Statistical Analysis

All statistical analysis and visualization were conducted by R (version 3.6.3). Kaplan-Meier analysis was carried out using the survival package, correlation heatmap was visualized by ggplot2 package, radar chart was illustrated by ggradar and ggplot2 package. Tumor purity is a major confounding factor in this analysis, since most immune cell types are negatively correlated with tumor purity. Therefore, we use the partial spearman’s correlation to perform purity adjustment association analysis, and explore the relationship between infiltrates estimation value and SERPINH1 expression. *p* < 0.05 was considered statistically significant.

## Results

### SERPINH1 Is Abnormally Expressed in Human Pan-Cancer

**FIGURE 1:**
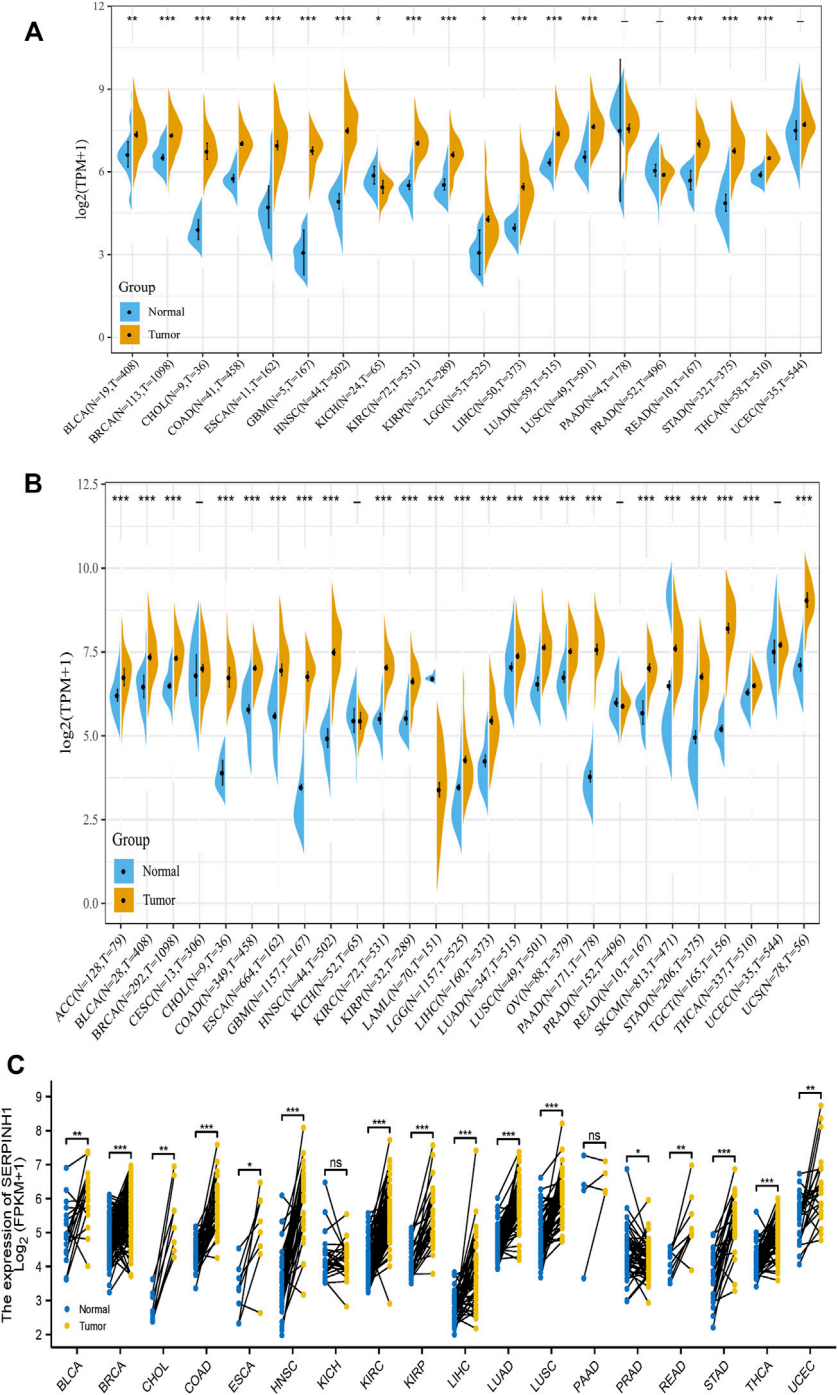
SERPINH1 is abnormally expressed in human pan-cancer. **(A)** Differential expression of SERPINH1 in cancers and normal tissues from the TCGA dataset. **(B)** Data from TCGA and GTEx dataset showed differential expression of SERPINH1 in mutiple cancers. **(C)** Differential expression of SERPINH1 in cancers and normal tissues from TCGA dataset (paired samples, *n* = 11,093). *p < 0.05, **p < 0.01, ***p < 0.001.

In this study, we aimed to analysis the expression of SERPINH1 across various cancer types. Based on the TCGA database, we found that SERPINH1 expression is relatively higher in BRCA, CHOL, COAD, ESCA, GBM, HNSC, KIRP, LIHC, LUAD, LUSC, READ, STAD, and THCA (all *p* < 0.001), BLCA (*p* < 0.01), and LGG (all *p* < 0.05) than those in nomal tissues (Figure 1A). Taking into account the lack of normal samples in the TCGA database for some cancer types, we integrated the GTEx database for further analysis and the results showed that the expression level of SERPINH1 in the tumor tissues of ACC, BLCA, BRCA, CHOL, COAD, DLBC, ESCA, GBM, HNSC, KIRC, KIRP, LAML, LGG, LIHC, LUAD, LUSC, OV, PAAD, PRAD, READ, SKCM, STAD, TGCT, THYM, and UCS (all *p* < 0.001); UCEC (*p* < 0.01); and SARC, THCA (all *p* < 0.05) are much higher than the corresponding control tissues (Figure 1B). In paired samples, the expression of SERPINH1 in tumor tissues of BRCA, COAD, HNSC, KIRC, KIRP, LIHC, LUAD, LUSC, STAD, and THCA (all *p* < 0.001); BLCA, CHOL, and KICH (all *p* < 0.01); and ESCA, READ, and UCEC (all *p* < 0.05) are significantly higher than the corresponding control tissues (Figure 1C). The above results showed that SERPINH1 expressed abnormally across multiple cancers. Interestingly, we noticed that in PRAD, compared with control tissues, the expression of SERPINH1 has no significant difference in independent samples, but there is a significant difference in paired samples (*p* < 0.05).

### Prognostic Potential of SERPINH1 in Human Pan-Cancer

**FIGURE 2:**
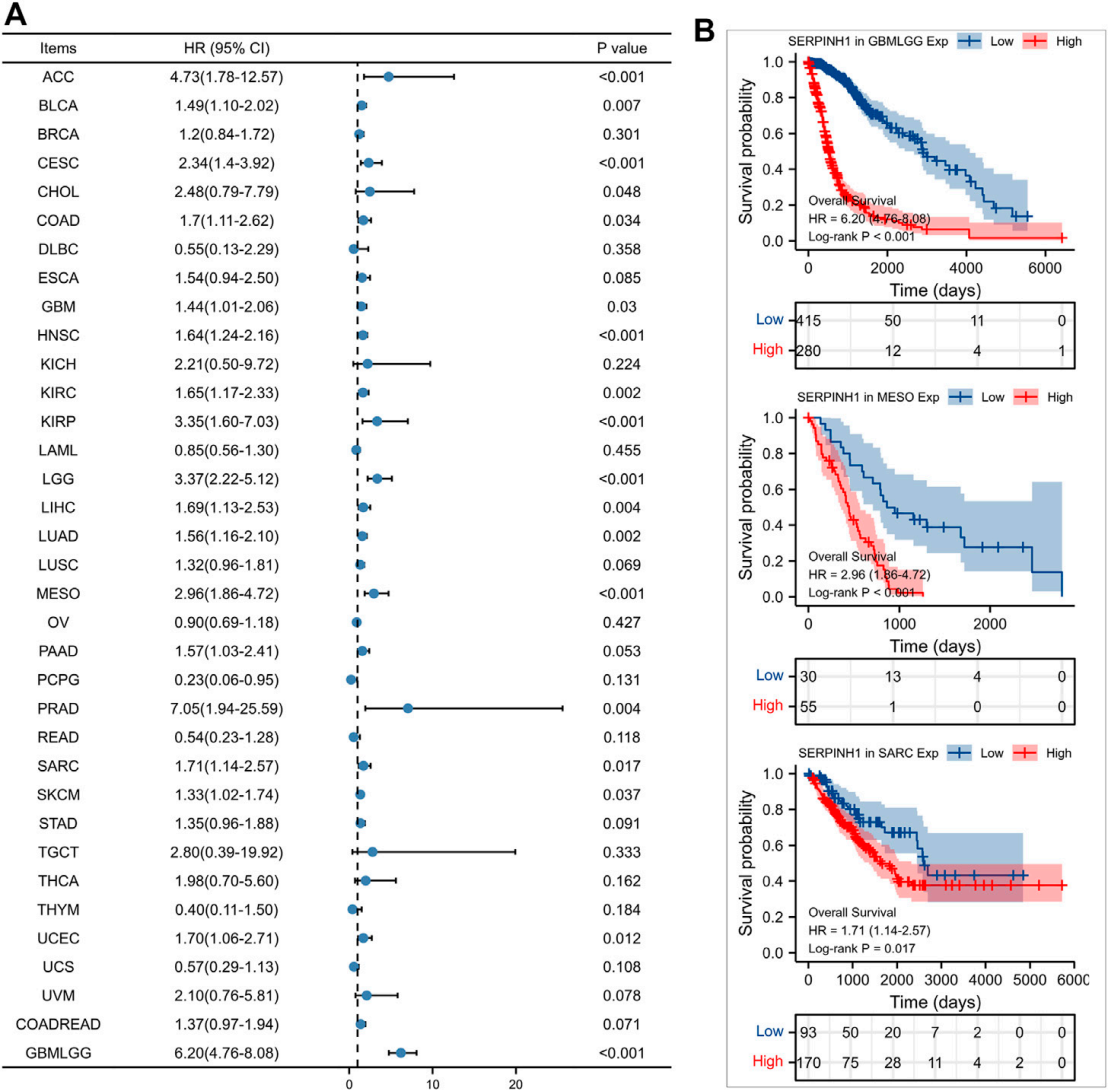
Relationship of SERPINH1 expression level with patients’ OS. **(A)** Forset plot of hazard ratio of SERPINH1 in human pan-cancer. **(B)** Kaplan-Meier OS curves for patients stratified by different expression levels of SERPINH1 in three cancer types.

**FIGURE 3:**
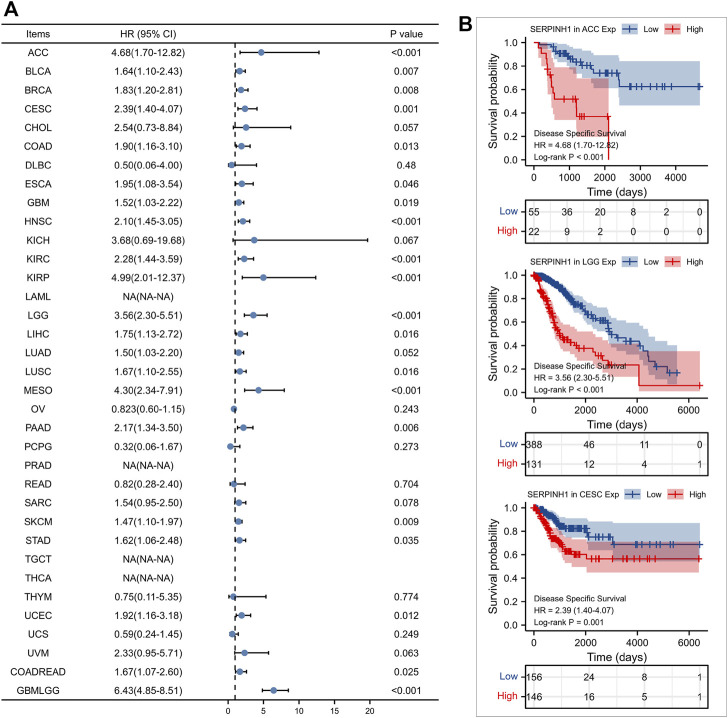
Relationship of SERPINH1 expression level with patients’ DSS. **(A)** Forset plot of hazard ratio of SERPINH1 in human pan-cancer. **(B)** Kaplan-Meier DSS curves for patients stratified by different expression levels of SERPINH1 in three cancer types.

**FIGURE 4:**
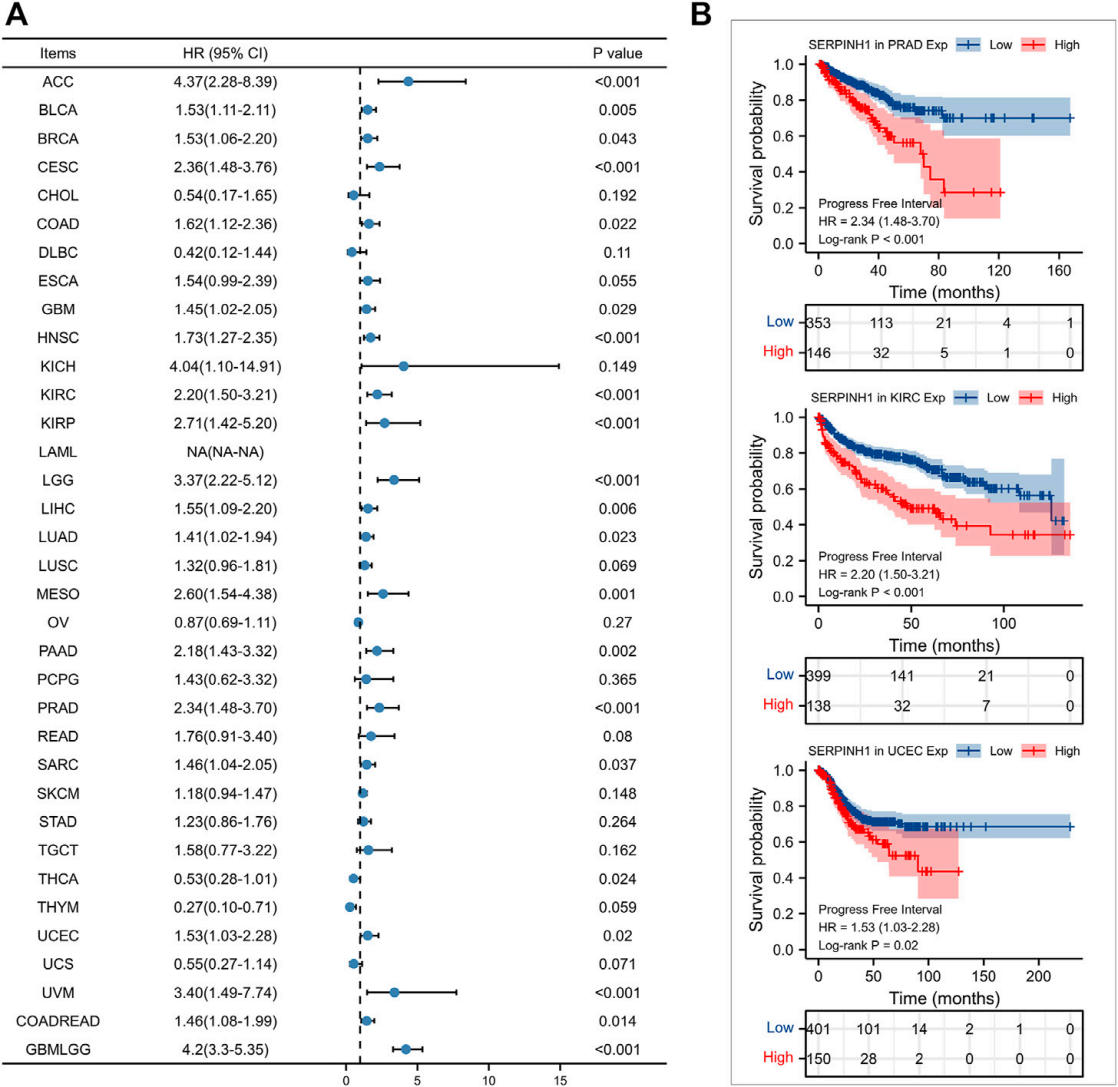
Relationship of SERPINH1 expression level with patients’ PFI. **(A)** Forset plot of hazard ratio of SERPINH1 in human pan-cancer. **(B)** Kaplan-Meier PFI curves for patients stratified by different expression levels of SERPINH1 in three cancer types.

Furtherly, we evaluated the relationship between the expression of SERPINH1 and the prognosis of pan-cancer patients. According to the Log-rank analysis, we found that in 33 cancers, the high expression of SERPINH1 significantly reduces the OS in 18 types of cancers including ACC, BLCA, CESC, CHOL, COAD, GBM, HNSC, KIRC, KIRP, LGG, LIHC, LUAD, MESO, PRAD, SARC, SKCM, UCEC, and GBMLGG (Figures 2A,B). Since there may be non-tumor death factors during follow-up, we further explored the relationship between SERPINH1 expression and patients’ DSS. Results indicated that high expression of SERPINH1 significantly impacted DSS in 20 types of cancers including ACC, BLCA, BRCA, CESC, COAD, ESCA, GBM, HNSC, KIRC, KIRP, LGG, LIHC, LUSC, MESO, PAAD, SKCM, STAD, UCEC, COADREAD, and GBMLGG (Figures 3A,B). In addition, we also explored the correlation of SERPINH1 with patients’ PFI, which indicated that high expression of SERPINH1 unfavorably impacted PFI in 21 types of cancers including ACC, BLCA, BRCA, CESC, COAD, GBM, HNSC, KIRC, KIRP, LGG, LIHC, LUAD, MESO, PAAD, PRAD, SARC, THCA, UCEC, UVM, COADREAD, and GBMLGG (Figures 4A,B).

### PPI Network Construction and Functional Enrichment Analysis of SERPINH1

**FIGURE 5:**
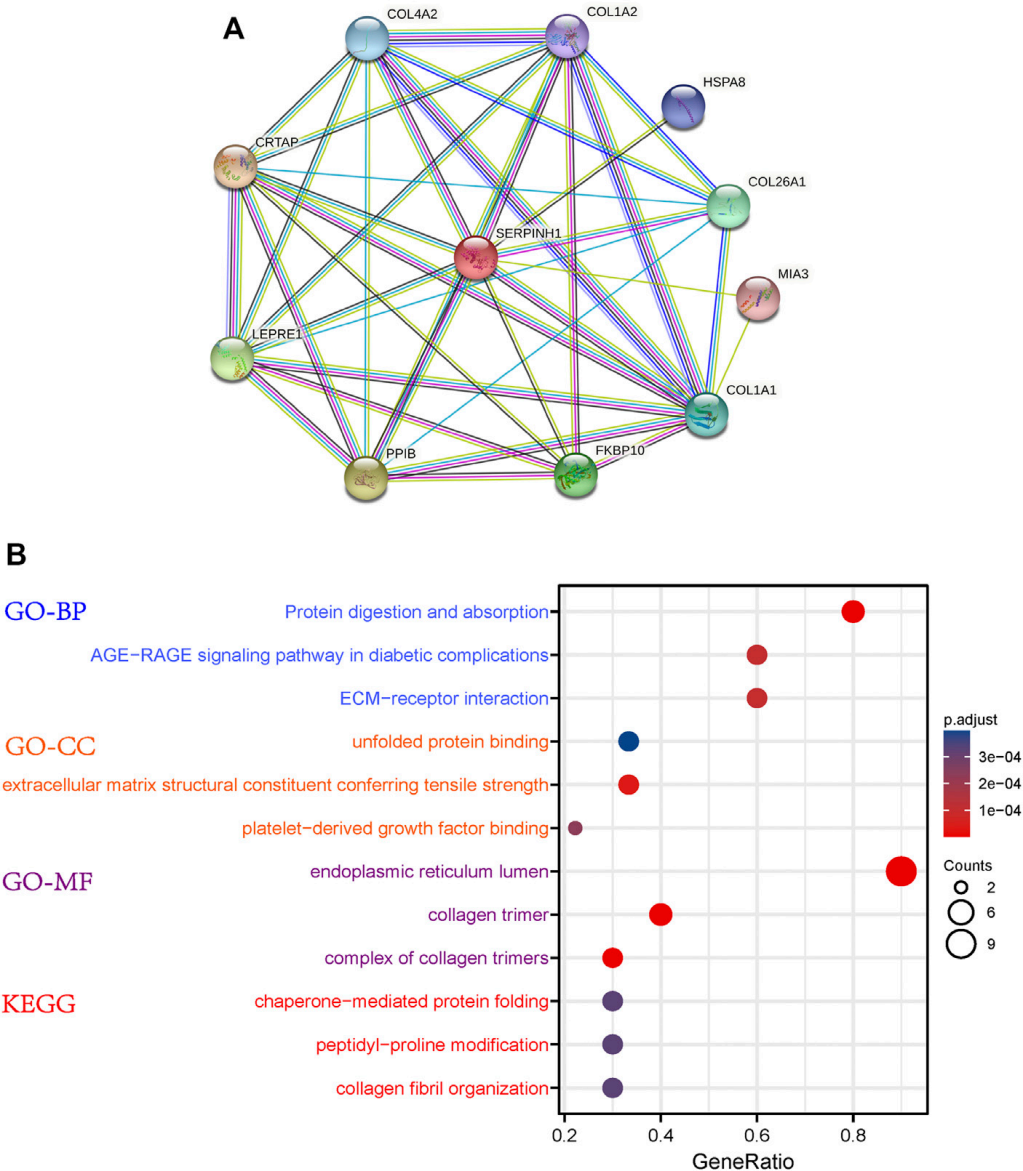
Protein-protein interaction (PPI) network construction and enrichment analysis. **(A)** PPI for SERPINH1. **(B)** GO and KEGG enrichment analysis for SERPINH1.

**FIGURE 6:**
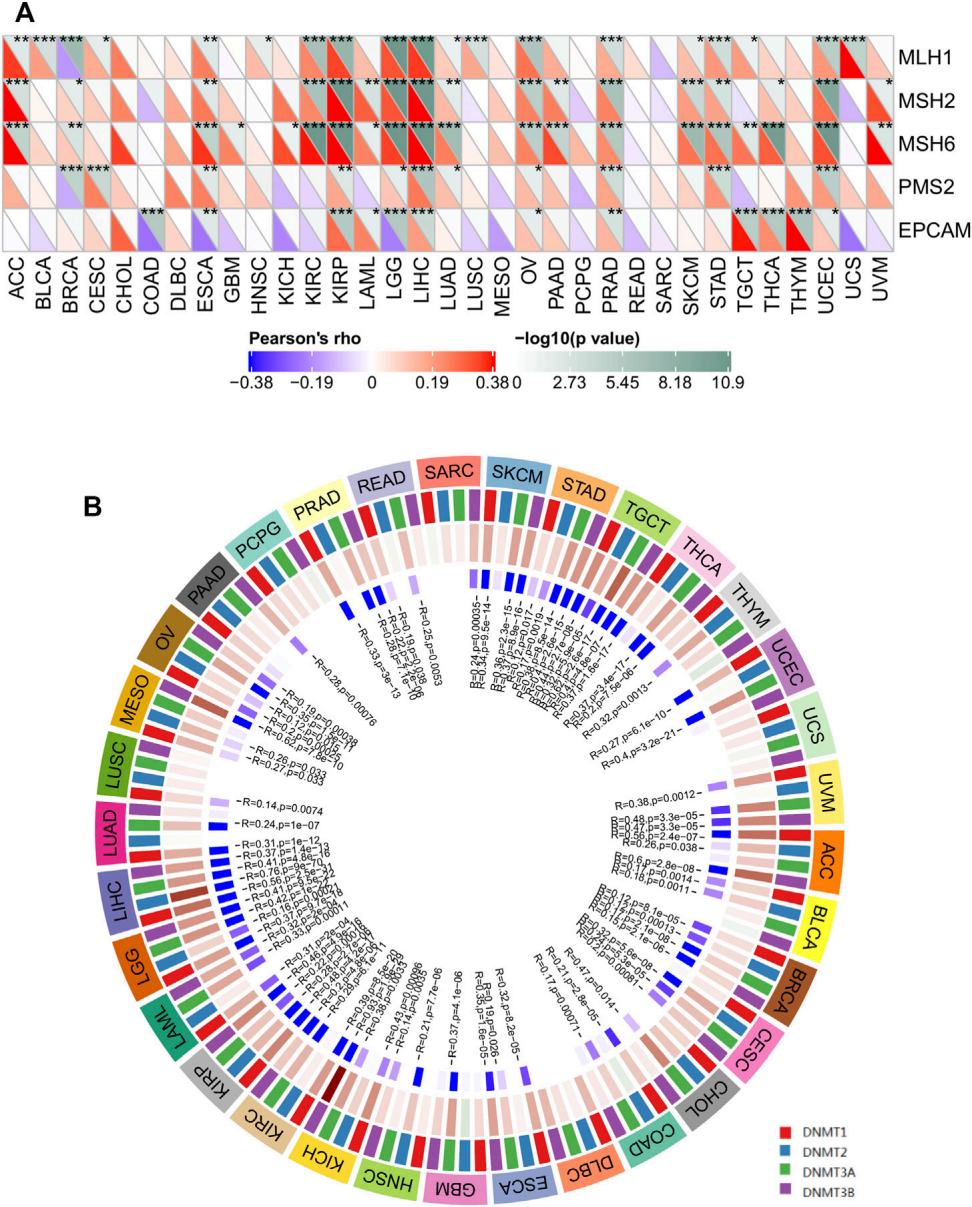
The correlations of SERPINH1 expression with five MMR genes and four DNA methyltransferases in human pan-cancer. **(A)** The correlation of SERPINH1 expression with expression levels of five MMR genes across cancers. **(B)** The correlation of SERPINH1 expression with four DNA methyltransferases across cancers. *p < 0.05, **p < 0.01, ***p < 0.001.

The PPI network of SERPINH1 was shown in Figure 5A. The results of GO enrichment analysis showed that SERPINH1 significantly focused on protein digestion and absorption, AGE-RAGE signanling pathway in diabetic complications, ECM-receptor interaction (biological process, BP); unfolded protein binding, extracellular matrix structural constituent conferring tensile strength (cell components, CC); and endoplasmic reticulum lumen, collagen trimer (molecular function, MF); According to the *p*-value, the top three items from the three categories were selected to plot a bubble (Figure 5B). KEGG enrichment analysis (Figure 6B) indicated that SERPINH1 was significantly enriched with chaperone-mediated protein folding and peptidyl-proline modification *etc*.

### Correlation Between SERPINH1 Expression and TMB or MSI in Human Pan-Cancer

**FIGURE 7:**
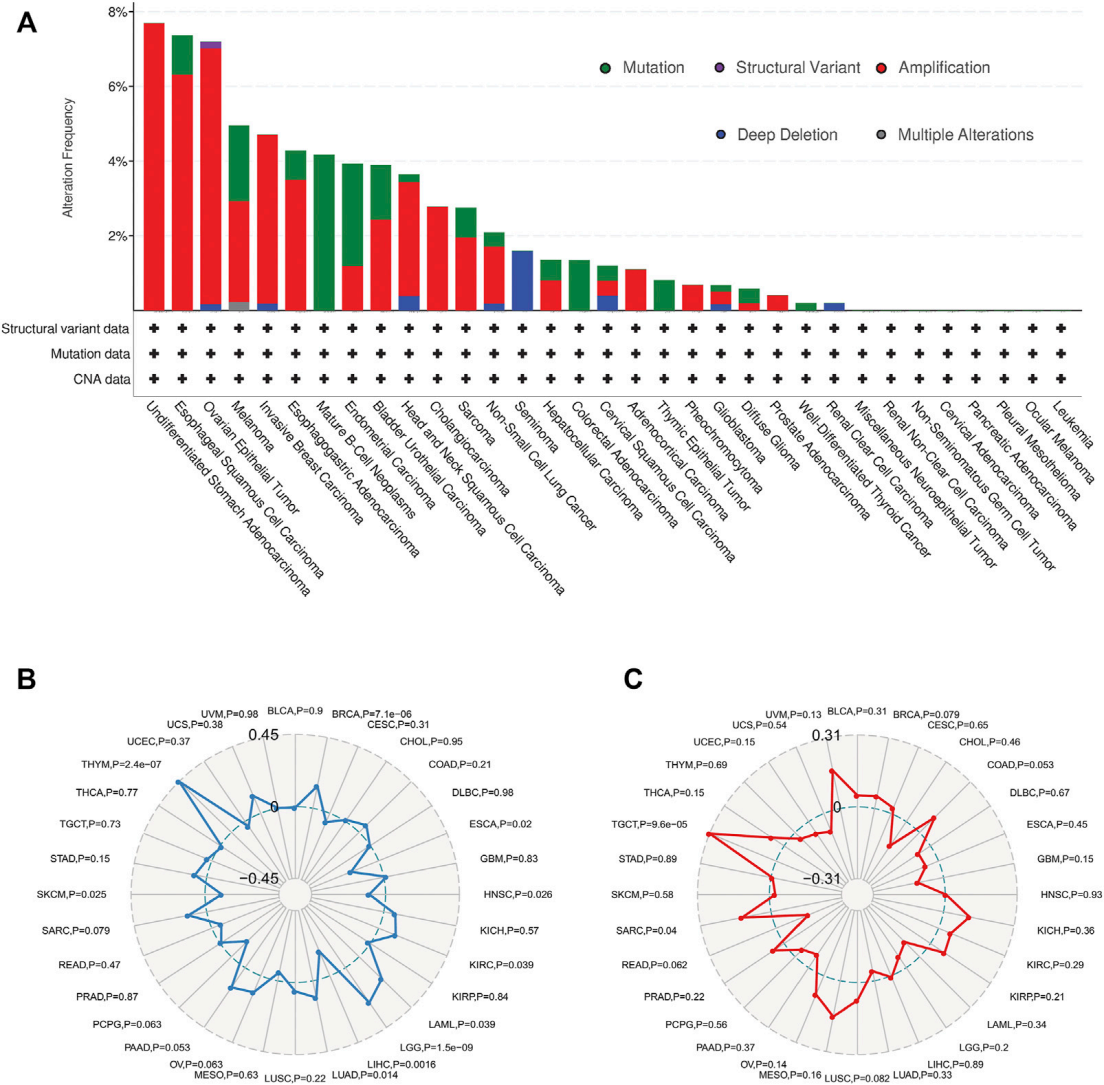
Overview of SERPINH1 mutation information and correlations with TMB and MSI in human pan-cancer. **(A)** SERPINH1 mutations in 30 cancer types. **(B)** The correlations of SERPINH1 expression with TMB in human pan-cancer. **(C)** The correlations of SERPINH1 expression with MSI in human pan-cancer.

The genetic alteration status of SERPINH1 in different cancer samples were observed via the TCGA cohorts. As shown in Figure 7A, the highest alteration frequency of SERPINH1 is “amplification” type, accounting for 7% of 13 patients with undifferentiated STAD. The “mutation” was the only type in 48 patients with mature B-cell neoplasms, which show an alteration frequency of 4.17%. 63 seminoma cases with genetic alteration (1.59% frequency) had copy number deletion of SERPINH1.

Tumor mutation burden (TMB), which counts the number of somatic mutations per megabase (mut/Mb), is an emerging potential biomarker for immunotherapy (Aggarwal et al., 2020). Microsatellite instability (MSI) is closely association with the development of most cancers (Matsuno et al., 2019). Here, we evaluated the correlation of SERPINH1 expression with TMB and MSI. Results showed that SERPINH1 expression was positively related to TMB in BRCA, KIRC, LAML, LGG, LUAD, and THYM; while negatively associated with TMB in ESCA, HNSC, LIHC, and SKCM (Figure 7B). In terms of MSI, the SERPINH1 expression was positively related to MSI in SARC, and TGCT (Figure 7C).

### SERPINH1 Is Associated With MMR Gene and DNA Methylation Across Cancers

Correct replication of the genome is a prerequisite for the maintenance of genome stability (Barra and Fachinetti, 2018). MMR serves as an important factor in maintaining genome stability in response to spontaneous DNA damage. MMR-deficient cells usually show microsatellite instability but display low levels of genome instabilities (Jia et al., 2017). To determine the role of SERPINH1 in tumor progression, we evaluated the association of the SERPINH1 expression with five MMR genes mutation levels. As results shown in Figure 6A, SERPINH1 expression was highly related to five MMR genes in seven cancers, including ESCA, KIRP, LGG, LIHC, OV, PRAD, and UCEC.

Global DNA hypomethylation and focal DNA hypermethylation are associated with tumorigenesis. Methylation of DNA is catalyzed by the DNA methyltransferase (DNMT) family of enzymes, with DNMT3A and DNMT3B catalyzing *de novo* DNA methylation and DNMT1 mediating both *de novo* and maintenance methylation of DNA (Suppiah et al., 2019). We therefore sought to determine whether SERPINH1 influences methyltransferases -mediated DNA repair, then we evaluated the relationships between SERPINH1 and four DNMTs. As shown in Figure 6B, SERPINH1 expression was highly associated with these four DNMTs in multiple cancers, specifically in ACC, BLCA, BRCA, CESC, COAD, ESCA, GBM, HNSC, KICH, KIRP, KIRC, LAML, LGG, LIHC, MESO, OV, PRAD, SKCM, STAD, TGCT, THCA, and UVM. Results indicate that SERPINH1 may regulate the tumor progress by MMR-mediated DNA repair and DNA methylation.

### Relationship Between SERPINH1 Expression and Immunoregulators in Human Pan-Cancer

**FIGURE 8:**
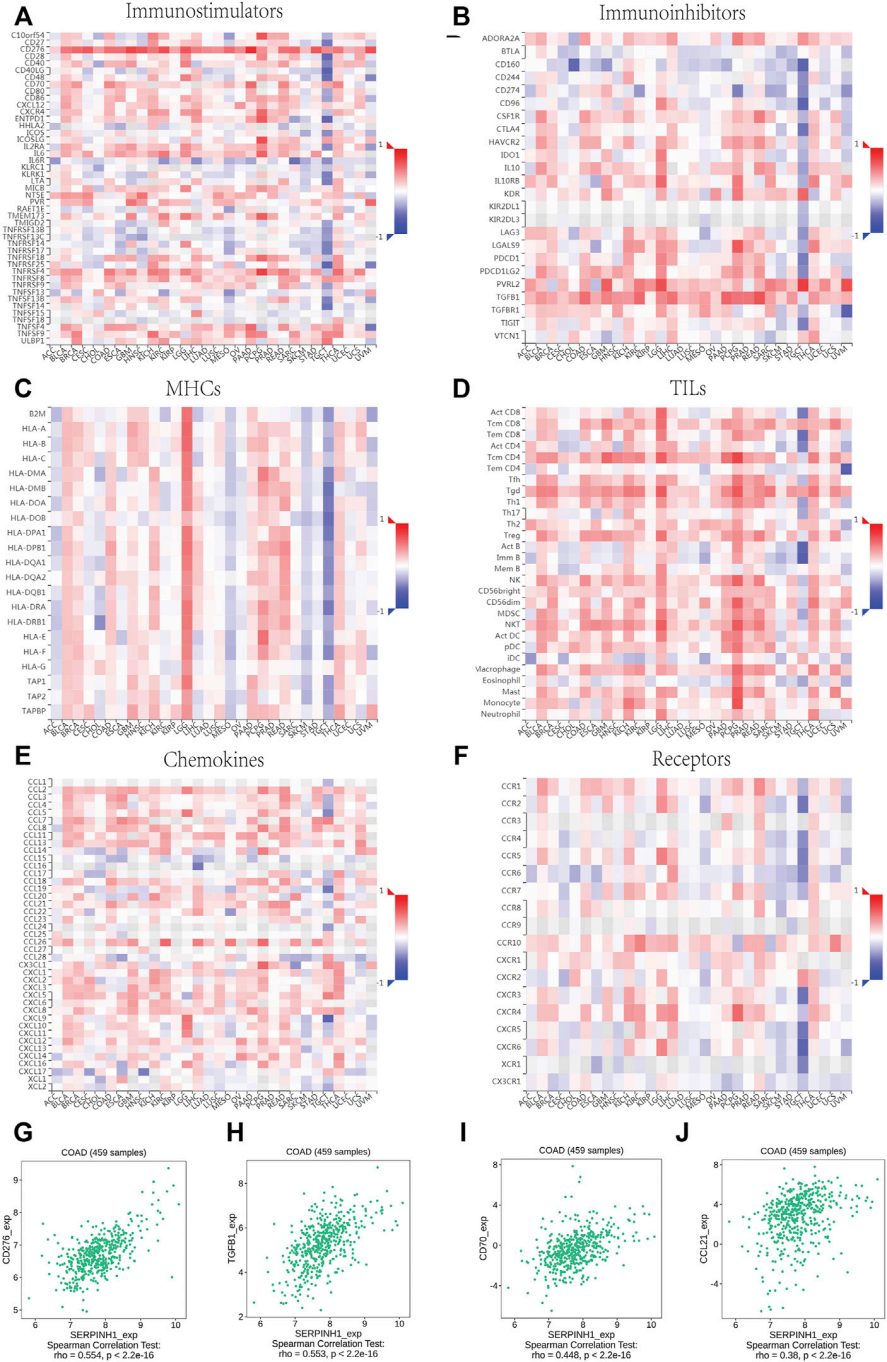
Relationship of SERPINH1 expression and immunoregulators. **(A–F)**: Spearman correlations between immunostimulators, immunoinhibitors, MHC molecules, lymphocyte (TILs), receptors, and chemokines respectively across multiple types of human cancers. **(G–J)**: The correlation between the expression of SERPINH1 and the expression of CD276, TGFB1, CD70, and CCL21, respectively, in COAD.

The relationship between SERPINH1 and immunomodulators were shown in Figures 8A–F). Take COAD for example, we founded that SERPINH1 was significant positive correlation with several immunomodulators, including immunostimulator CD 276 and CD70 expressions (spearman correlation of 0.554 and 0.448 respectively), immunoinhibitor TGFB1 expression (spearman correlation of 0.553), and CCL21 expression (spearman correlation of 0.38) (Figures 8G–I). Based on the results, we can infer that the immunomodulators CD 276, TGFB1, CD70, and CCL21 might be regulated by SERPINH1 in COAD. Thus it can be seen that SERPINH1 interacts with immune regulation and may become a potential biomarker which has an important impact on the development of cancers and prognosis of patients.

**FIGURE 9:**
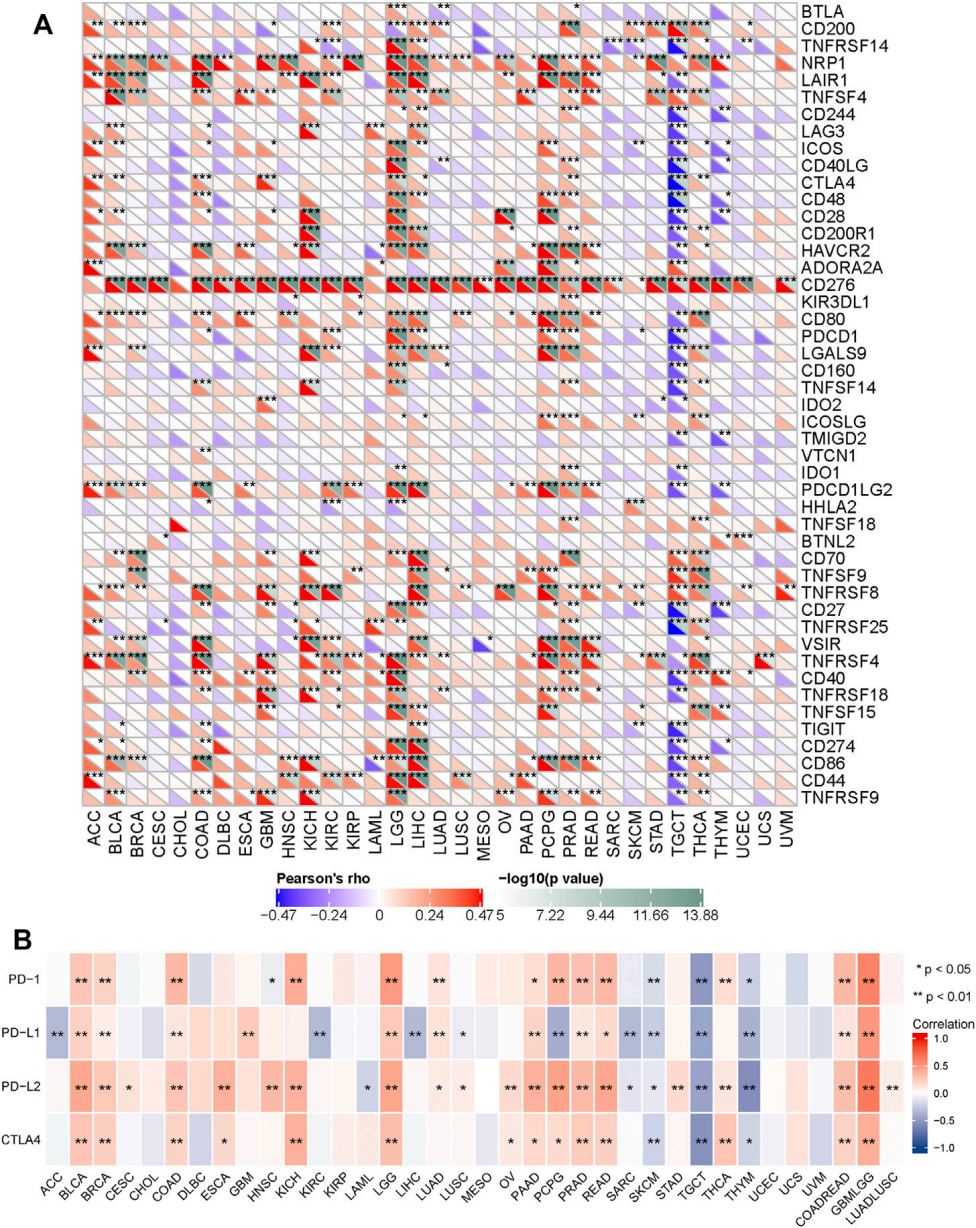
Correlation of SERPINH1 with common immunue checkpoints and immunotherapy targets. **(A)** SERPINH1 is correlated with 47 common immunue checkpoints across human cancers. **(B)** SERPINH1 is correlated with immunotherapy targets across human cancers. ^*^ *p* < 0.05, ^**^ *p* < 0.01, ^***^ *p* < 0.001.

Furtherly, we performed a correlation analysis to explore the relationship between SERPINH1 and 47 common immune checkpoint genes. Results showed that SERPINH1 expression correlated with 38 immune checkpoint genes in TGCT, and 35 immune checkpoint genes in LGG (Figure 9A). The relationship between SERPINH1 and immunotherapy targets (PD-1, PD-L1, PD-L2, and CTLA4) was shown in Figure 9B. Results indicated that SERPINH1 was positively correlated with immunotherapy targets in BLCA, BRCA, COAD, LGG, PAAD, PCPG, PRAD, READ, COADREAD, and LUADLUSC; and negatively correlated with immunotherapy targets in SKCM, TGCT, and THYM.

**FIGURE 10:**
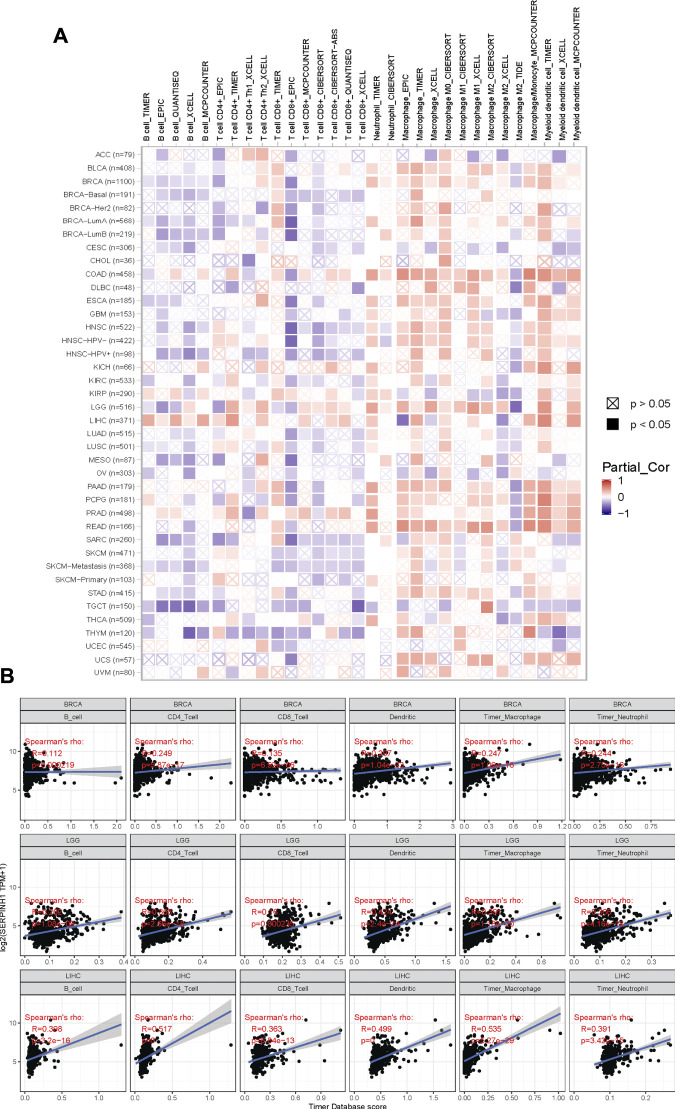
Correlation of SERPINH1 with immune infiltration level in human pan-cancer. **(A)** SERPINH1 expression is correlated with infiltrating immune cells across human cancers. **(B)** SERPINH1 expression is positively correlated with immune infiltration in BRCA, LGG, and LIHC.

The tumor microenvironment consists of tumor cells, infiltrating immune cells, and stromal cells (Zhang et al., 2020). We utilized the TIMER database to explore potential associations between SERPINH1 and the infiltrating immune cells across human cancers (Figure 10A). This study focuses on showing the correlation between SERPINH1 expression and six immune infiltrating cells, including B cell, CD4^+^ T cell, CD8^+^ T cell, dendritic cell macrophage cell and neutrophil cell in BRCA, LGG, and LIHC (Figure 10B). From the above results, we can infer that SERPINH1 acts significantly in tumor immunity.

### Validation for SERPINH1 by Immunohistochemistry

**FIGURE 11:**
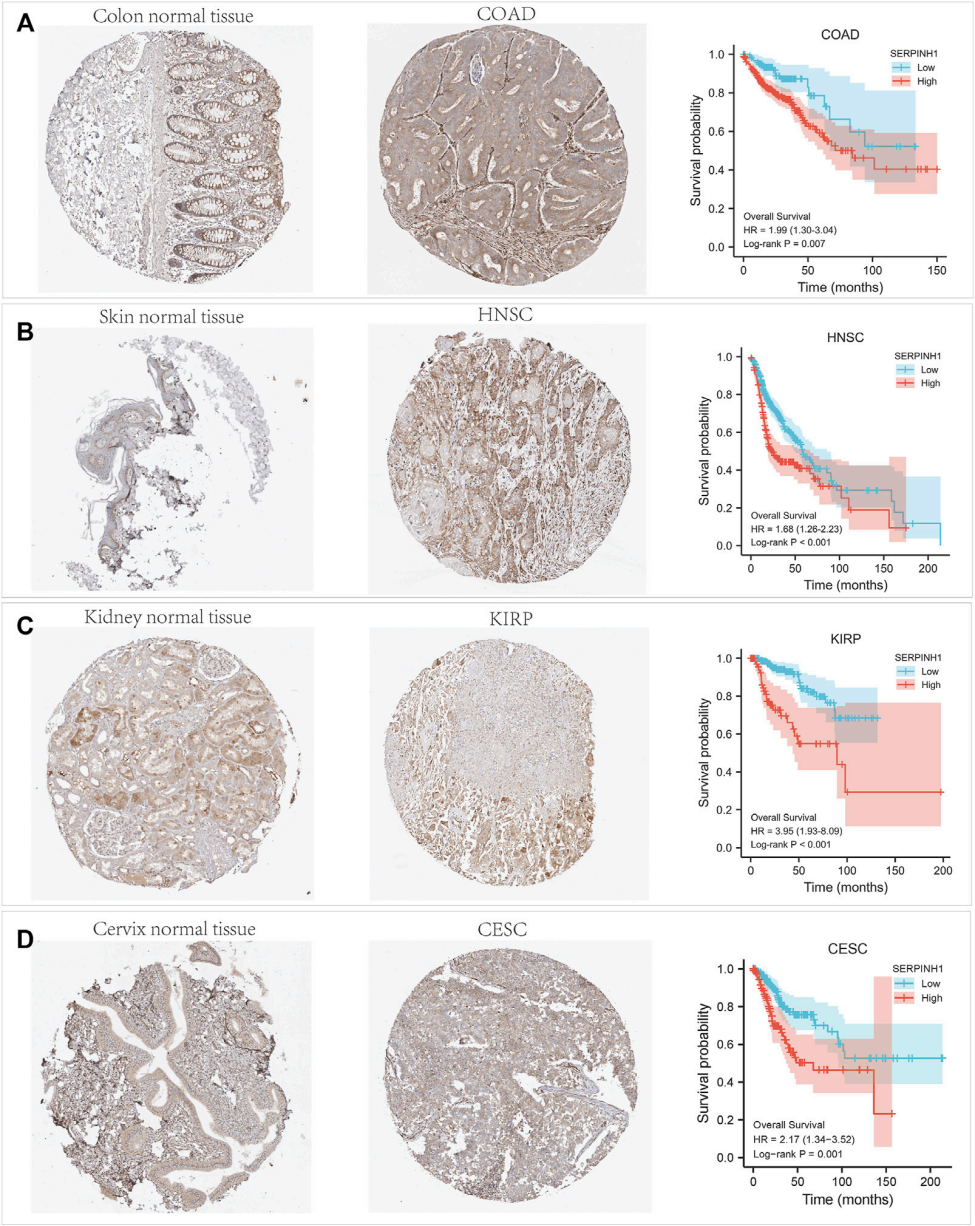
Immunohistochemistry (IHC) staining of SERPINH1 based on the Human Protein Atlas. **(A)** IHC staining of SERPINH1 in colon normal tissue and COAD, Kaplan-Meier OS curves for patients stratified by different expression levels of SERPINH1 in COAD. **(B)** IHC staining of SERPINH1 in skin tumor tissue and HNSC, Kaplan-Meier OS curves for patients stratified by different expression levels of SERPINH1 in HNSC. **(C)** IHC staining of SERPINH1 in kidney normal tissue and KIRP, Kaplan-Meier OS curves for patients stratified by different expression levels of SERPINH1 in KIRP. **(D)** IHC staining of SERPINH1 in cervix tumor tissue and CESC, Kaplan-Meier OS curves for patients stratified by different expression levels of SERPINH1 in CESC.

The immunohistochemistry data of SERPINH1 was acquired from the Human Protein Atlas database (http://www.proteinatlas.org) for validation. As shown in Figure 11, SERPINH1 staining was higher in tumor samples than in normal tissue, which was consistent with the result of survival analysis, indicating that high expression of SERPINH1 is a risk factor in COAD, HNSC, KIRP, and CESC.

## Discussion

Serpins (serine-protease inhibitors) are a superfamily of proteins that share a conserved tertiary structure (Choi et al., 2019). Among 16 phylogenetic clades, SERPINH1 localized to the endoplasmic reticulum and plays a role in collagen biosynthesis as a collagen-specific molecular chaperone. Alternatively spliced transcript variants have been observed for this gene, and a pseudogene of this gene is located on the short arm of chromosome 9.

In this study, the exploratory analysis of SERPINH1 in pan-cancers was carried out in the TCGA database, and the results showed that in both independent samples and paired samples, SERPINH1 were highly expressed in a variety of cancers (including BLCA, BRCA, CHOL, COAD, ESCA, HNSC, KIRC, KIRP, LIHC, LUAD, LUSC, READ, STAD, and UCEC) compared with normal tissues. We further analyzed whether the high expression of SERPINH1 was related to the poor prognosis of pan-cancer, and the results showed that high expression of SERPINH1 significantly reduced the OS, DSS, and PFI in 11 types of cancers including ACC, BLCA, CESC, COAD, GBM, HNSC, KIRC, KIRP, LGG, LIHC, and MESO.

TMB is an emerging biomarker in cancer characterized by MSI. TMB has been described as a powerful predictor of tumor behavior and response to immunotherapy (Chan et al., 2019; Chen et al., 2019). In this study, we observed the genetic alteration status of SERPINH1 in different cancers and evaluated the correlation of SERPINH1 expression with TMB and MSI. Results showed that SERPINH1 was positively related to TMB in BRCA, KIRC, LAML, LGG, LUAD, and THYM; while negatively associated with TMB in ESCA, HNSC, LIHC, and SKCM. In terms of MSI, the SERPINH1 expression was positively related to MSI in SARC and TGCT.

SERPINH1 encodes HSP47, a chaperone located in the endoplasmic reticulum that appears to preferentially recognize and help maintain the folded state of the type I procollagen trimer (Macdonald and Bächinger, 2001). Two osteogenesis imperfecta mutations have been reported in SERPINH1, one in dachshunds (Drögemüller et al., 2009) and one in human moderately severe osteogenesis imperfecta (Duran et al., 2015 and Christiansen et al., 2010). In both instances, the homozygosity of the missense mutation corresponding main functional domain of SERPINH1 was determined, and the serine-type endopeptidase inhibitor domain recognizing Xaa–Arg–Gly-containing procollagen sequences were confirmed (Ono et al., 2012). However, the mutations and mutation sites of SERPINH1 in cancer are rarely reported. In this study, we investigated the SERPINH1 mutation information and evaluated the association of the SERPINH1 with five MMR and four DNMTs genes. Results showed that the highest alteration frequency of SERPINH1 was 7.69% in STAD, and 7.37% in ESCA. In addition, SERPINH1 was highly related to five MMR and four DNMTs genes in 7 and 22 cancers, respectively, which indicated that the mutant SERPINH1 may play a key role in the occurrence and prognosis of patients with related cancers.

Tumor immune microenvironment is known to play a pivotal role in the occurrence and development of cancer (Yu et al., 2019). Considering that the up-regulation of SERPINH1 expression level was related to shorter OS, DSS and PFI, we speculated that SERPINH1 may involved in tumor immune response. Here, we used the TIMER database to verify the hypothesis. The results showed that SERPINH1 was related to the immunostimulators, immunoinhibitors, MHC molecules, TILs, receptors, and chemokines in multiple types of human cancers. We further found the co-expression of SERPINH1 with immune checkpoint markers across cancers, specifically in TGCT (38/47), LGG (35/47), and PRAD (32/47). Tumor-infiltrating immune cells are considered to be a marker of host antitumor immune response and prognostic feature (Noman et al., 2020; and; Väyrynen et al., 2020). CD8^+^ T-cells and NK cells play a predominant role in antitumoral immune response via immune checkpoints (Titov et al., 2021). In our present study, we found that SERPINH1 was significantly correlated with six immune infiltrating cells including B cell, CD4^+^ T cell, CD8^+^ T cell, dendritic cell macrophage cell and neutrophil cell in multiple cancers, particularly in BRCA, LGG, and LIHC. All this results infer that SERPINH1 may recruit and regulate infiltrating immune cells to inhibit or promote the progression of cancers, which strongly suggest that SERPINH1 serves as a key factor in cancer immunity.

In summary, this study revealed that SERPINH1 was highly expressed and related to poor prognosis in a variety of cancers, especially in BLCA, COAD, HNSC, KIRC, KIRP, and LIHC. Furthermore, SERPINH1 expression was found to be associated with MMR, MSI, TMB, and DNA methylation in multiple cancers. In addition, SERPINH1 was correlated with multiple immunoregulators and immune infiltration level in a variety of cancers, particularly in BRCA, LGG, and LIHC. To conclude, SERPINH1 may play a important role as a prognostic biomarker for human pan-cancer and the results of this study may provide a novel and effective immunological antitumor strategy for tumor immunity research. Since this research was based on data analysis, further experimental verification needs to be carried out.

## Data Availability

The datasets presented in this study can be found in online repositories. The names of the repository/repositories and accession number(s) can be found in the article/Supplementary Material.
